# Nile Ward PICU Violence Reduction Quality Improvement Project - One Year on

**DOI:** 10.1192/bjo.2022.330

**Published:** 2022-06-20

**Authors:** Mehtab Rahman, Claudia Taylor, Roda Abdullahi, Anthony Okwuokei, Mohammed Kaji, Matthew Waugh, Biganani Magadlela, Jessica Coplestone, Ruby Fell

**Affiliations:** College of North West London, London, United Kingdom

## Abstract

**Aims:**

To reduce incidents of inpatient violence and aggression at Nile Ward Psychiatric Intensive Care Unit (PICU), St Charles Hospital by at least 30% between December 2019 and December 2021. Reducing inpatient violence is a major quality improvement (QI) priority for CNWL NHS Foundation Trust.

**Methods:**

Nile Ward refined a number of their successful change ideas within this project and a number of new innovative ideas were tested and successfully implemented as part of the Violence Reduction QI Project:

1. Improved risk assessment tool: Risk assessment tool to predict/manage violence in the ward was further improved using evidence based observation and best practice recommendations over the course of 2021.
2. Brand new Staff Photo Board: Regularly updated photoboard with non-hierachical list of all staff.
3. Patient Feedback Board: Patient experience, comments and feedback displayed in common areas.
4. Co-produced Mutual Expectations: A set of expectations created in co-production with patients displayed in the communal areas of the ward to be followed by both staff and patients.
5. Gardening sessions: A safe socially distanced space for patients to be involved in growing and caring for the Nile Ward garden with our Activities Coordinator, including a brand new herb garden.
6. Tailored Physical Fitness Programmes: Focus on physical activity through garden fitness sessions and 1–1 fitness sessions in the gym. Average weight gain for patients has declined from 4.4 kg to 2.8 kg (39% reduction) during hospital stay. Tailored physical fitness sessions created for patients who are frail, diabetic or have significant cardiometabolic risk factors.
7. Celebrating Diversity: Special events hosted throughout the year to celebrate diversity and promote tolerance.
8. Enhanced Clinical Reviews: Consultant led patient reviews every weekday to optimise treatment and enable quick recovery using a multidisciplinary, holistic, trauma informed approach.
9. Weekly Cooking Sessions: Patient led cooking sessions using healthy ingredients every week. The food is eaten as a communal meal by patients and staff. A ‘Friday Fry-Up’ takes place monthly where patients and staff share a health fry-up in the ward's dining area.
10. Mindfulness Meditation: A QI intervention introduced to embed mindfulness and meditation as core therapeutic intervention to improve emotional regulation and to reduce violence.
11. Triangle of Care: Carers strongly encouraged to attend ward rounds and care planning from the very beginning of a patient's journey at Nile Ward using a triangle of care approach.

**Results:**

Between December 2019 - December 2020, Nile Ward reduced violence in the ward by 35% and the MDT continued to make further innovations to reduce violence further, as demonstrated in this poster.

Between December 2020 - December 2021, Nile Ward reduced violence in the ward by 51%.

Further details about the results will be published in the poster.

**Conclusion:**

Nile Ward has successfully implemented innovative interventions using a QI methodology to successfully reduce the level of violence and serious incidents in the ward by 51%. The number of rapid tranquillisations and use of restrictive interventions such as restraints has reduced significantly. Our patients are able to recover in a safe environment and their feedback is testament to their positive patient experience during their inpatient stay. Reduced verbal and physical assaults on staff have improved staff confidence, retention, well-being and overall satisfaction. Our work has been recognised internationally through the delivery of keynote presentations at conferences National Association of Intensive Care Unit (NAPICU) National Conference 2021 & the Royal College of Psychiatrists National QNPICU Conference 2021 to discuss their Violence Reduction best practices with mental health teams in the United Kingdom and abroad.

